# Research on the influencing factors and mechanism of AR e-commerce consumers’ purchase intention

**DOI:** 10.1371/journal.pone.0351158

**Published:** 2026-06-16

**Authors:** Xiaodong Zhang, Pan Yang, Chunrong Guo, Pan He

**Affiliations:** 1 School of Economics and Management, Inner Mongolia Agricultural University, Hohhot, China; 2 School of Economics and Management, Ningbo University of Technology, Ningbo, China; 3 School of Food Science and Engineering, Inner Mongolia Agricultural University, Hohhot, China; MCC Boyd Tandon School of Business, INDIA

## Abstract

Against the backdrop of the digital economy and the increasing integration of virtual and physical consumption scenarios, augmented reality (AR) e-commerce reshapes the shopping experience by overlaying virtual information onto real-world contexts, thereby accelerating the industry’s shift from traffic-driven competition to experience-based competition. However, existing studies remain insufficient in explaining the underlying mechanism through which AR e-commerce influences consumers’ purchase intention, and the applicability of the stimulus–organism–response (S–O–R) framework in virtual–real integrated environments has not been adequately extended. Drawing on the S–O–R paradigm, this study conceptualizes four key AR-enabled characteristics—immersive virtual try-on, interactive product display, real-time fusion shopping, and personalized recommendation—as stimulus variables (S). Perceived value and brand attitude are modeled as organism variables (O), and purchase intention is treated as the response variable (R), forming an integrated conceptual model. Using survey data collected from 482 valid respondents, structural equation modeling (SEM) is employed to test the proposed relationships. The results indicate that all four AR stimuli indirectly enhance purchase intention by positively affecting perceived value and brand attitude. Among the stimulus dimensions, real-time fusion shopping exerts the strongest positive effect on perceived value (β = 0.290), whereas personalized recommendation shows the most pronounced effect on brand attitude (β = 0.295). In addition, perceived value and brand attitude play partial mediating roles, and brand attitude demonstrates a stronger direct effect on purchase intention (β = 0.449) than perceived value (β = 0.347). Theoretically, the present study systematically translates AR e-commerce technological attributes into stimulus dimensions within the S–O–R framework, thereby extending its explanatory boundary in virtual–real integrated settings and offering an integrated “technology–scenario–behavior” analytical lens. Practically, the findings provide actionable insights for e-commerce platforms and brand managers seeking to optimize AR-driven experience design and improve conversion outcomes.

## 1. Introduction

With breakthroughs in enabling technologies such as 5G, cloud computing, and artificial intelligence, the deep integration of augmented reality (AR) with e-commerce has increasingly addressed key limitations of traditional online shopping, particularly the lack of vivid sensory experience and the inability to conduct direct product trials [1]. By enhancing product visualization and interaction, AR-enabled e-commerce has the potential to alleviate persistent industry challenges, including high return rates and low conversion efficiency. In parallel, consumers’ demand for richer and less homogenized offline shopping experiences continues to grow. In the context of experience-oriented retail transformation, AR e-commerce overlays virtual product information onto real-world environments, providing immersive try-on and interactive product presentation, and has gradually emerged as a critical driver of experience-oriented transformation in the e-commerce industry [2].

Although prior research has recognized the influence of AR technologies on consumer behavior [3–5], several important gaps remain. First, the internal mechanism through which AR e-commerce shapes purchase intention is still insufficiently explained; in particular, existing studies have not clearly articulated how specific technological attributes are translated into purchase intention through consumers’ psychological perceptions. Second, while the stimulus–organism–response (S–O–R) framework has been widely adopted to examine the relationship between environmental stimuli and behavioral responses, its application to virtual–real integrated consumption contexts lacks systematic extension. Specifically, the distinctive technological characteristics of AR e-commerce have not been adequately theorized and operationalized as context-appropriate “stimulus dimensions” within the S–O–R paradigm. Third, extant studies tend to focus on the effects of single AR features in isolation, leaving the synergistic influence of multiple AR-enabled attributes underexplored.

To address these gaps, this study pursues three main objectives: (1) to develop a conceptual model linking AR e-commerce technological attributes (stimuli) to purchase intention (response) through consumers’ psychological perceptions (organism); (2) to empirically test the mediating roles of perceived value and brand attitude; and (3) to identify the differentiated effects of distinct AR-enabled technological attributes on consumers’ purchase intention. Using survey data collected via a structured questionnaire, we employ structural equation modeling (SEM) to validate the proposed relationships. By doing so, the present study extends the applicability of the S–O–R framework to emerging digital consumption scenarios and provides both theoretical insights and practical guidance for experience optimization and sustainable development in AR-enabled e-commerce.

## 2. Definition, characteristics, and models of AR E-commerce

### 2.1 Definition of AR e-commerce

AR e-commerce refers to an online shopping method that integrates Augmented Reality (AR) technology into e-commerce platforms. Using devices such as computers, smartphones, and large displays, AR-enabled platforms overlay virtual product information onto the physical world. AR technology allows consumers to intuitively evaluate a product’s appearance, size, and other attributes in relation to themselves or their environment. By enabling access to rich product information and real-time usage simulations, AR can substantially enhance the overall shopping experience.

### 2.2 Characteristics of AR e-commerce

(1) Immersive Virtual Try-On

A defining feature of AR technology is its ability to deliver immersive experiences. With AR-enabled functions, consumers can project virtual products—such as shoes, cosmetics, eyewear, and jewelry—onto corresponding parts of their bodies during online shopping. AR-enabled functions enable consumers to virtually try on products and intuitively perceive how the items would look in real life.

In 2020, Dior collaborated with Snapchat to launch a “virtual shoe try-on” feature, allowing consumers to experience new sneaker models without leaving home. Similarly, in 2022, Amazon’s fashion retail division introduced a “virtual try-on” service covering more than 1,500 products. Such virtual try-on experiences help establish a stronger connection between consumers and products by enabling immersive interactions. They also allow consumers to more accurately assess the compatibility of products with their personal use contexts [6,7].

(2) Interactive Product Display

Gabriel et al. (2023) [8] argue that the interactivity of AR e-commerce lies in its ability to integrate virtual products with real environments. Based on camera-captured scenes, AR overlays virtual product information to support interactive product visualization. Consumers can freely rotate, zoom in, and zoom out on 3D models, examine product details and functions, and engage in interactive operations with virtual items, thereby gaining a comprehensive understanding of the products. Yang and Lin (2024) [9] further emphasize that AR enables real-time interaction with virtual models, allowing consumers to perceive product characteristics more fully and conveniently. These interactive display features can significantly enrich consumers’ experiences and strengthen their purchase intention in AR-enabled e-commerce settings.

(3) Real-Time Fusion Shopping

AR e-commerce enables consumers to preview and select products such as home décor and furniture within their actual environments. Virtual items can be placed directly into real-world spaces, enabling consumers to evaluate product dimensions and spatial fit in real time. Consumers can also assess how the product’s appearance complements existing surroundings. Compared with traditional e-commerce, AR shopping offers stronger interactivity and real-time feedback [10], which can improve the accuracy of consumers’ online purchase decisions.

(4) Personalization

AR e-commerce provides consumers with highly personalized experiences [11]. Through AR shopping interfaces, consumers can customize their experiences by selecting different virtual products [12]. In addition, AR systems can generate personalized product recommendations based on consumer preferences, browsing habits, style-matching data, and purchase histories. Compared with traditional e-commerce, such personalized services can reduce consumers’ search effort, help them quickly find suitable products, help them identify suitable products more efficiently, and ultimately improve the effectiveness of online shopping.

### 2.3 Models of AR e-commerce

AR-based online shopping has introduced new dynamics into e-commerce. It reduces barriers between consumers and platforms, reshapes the channels and modes of information acquisition, and enables the integration of virtual and real environments. By creating new forms of interaction between consumers and products, AR e-commerce has given rise to several representative application models, as summarized in Table 1.

**Table 1:** Main application models of AR e-commerce.

Application Case	Application Field	Main Function	Key Features
IKEA	Furniture	AR Furniture Placement Simulation:By leveraging AR technology, consumers can preview how furniture will look when placed in their homes. This helps effectively address issues of size and color mismatch, significantly reducing return and exchange rates.	Interactive Product Display, Real-Time Fusion Shopping, Personalization
Taobao, JD.com, Dewu, Shihuo, etc.	Shoes, Beauty Products	AR Virtual Shoe Try-On:Through AR-based interaction, shoes are superimposed onto the physical world, allowing consumers to see in real time how different styles, colors, and sizes look on their feet. This enhances the overall shopping experience. AR Virtual Makeup Try-On:AR technology enables online makeup trials, allowing consumers to easily test various products such as lipsticks, eyebrow pencils, and eyeliners on e-commerce platforms. With one click, they can complete a full virtual makeup look and evaluate different cosmetic options.	Immersive Virtual Try-On, Interactive Product Display, Real-Time Fusion Shopping, Personalization
Snapchat	Beauty Products, Shoes, Clothing, Glasses, and Other Products	AR Virtual Trial: By integrating AR technology with e-commerce, consumers can easily conduct online virtual try-on, makeup trials, and accessory fitting.	Immersive Virtual Try-On,Interactive Product Display,Real-Time Fusion Shopping,Personalization
Amazon	Beauty Products, Glasses, Shoes, Furniture, Appliances, etc.	AR Furniture, Appliance, and Toy Placement: Through AR-enabled functions, users can use their smartphone cameras to virtually place online products—such as furniture, appliances, and toys—within their own home environments.	Interactive Product Display,Real-Time Fusion Shopping,Personalization
Perfect Corp	Wristbands, Watches	AR Virtual Watch Try-On: AR Virtual Watch Try-On enables precise wrist size measurement while providing a dynamic view of the watch face, strap, and buckle. Consumers can enjoy a seamless 360° virtual try-on experience.	Immersive Virtual Try-On,Interactive Product Display,Real-Time Fusion Shopping,Personalization
WannaNails	Nail Art	AR Virtual Nail Try-On: AR Virtual Nail Try-On allows consumers to virtually try on hundreds of nail polish colors and instantly switch between various nail art designs. Selected products can be purchased instantly with a single click on the e-commerce platform.	Immersive Virtual Try-On,Interactive Product Display,Real-Time Fusion Shopping,Personalization

Source: Compiled by the authors based on related studies in [3,4,7,14], and [39].

## 3. Model construction of factors influencing consumer purchase intention in AR e-commerce

### 3.1 Theoretical background and literature review

#### 3.1.1 Theoretical foundation of the S–O–R framework.

The stimulus–organism–response (S–O–R) framework was originally proposed by Mehrabian and Russell (1974) [13]. The central logic of this theory posits that external environmental stimuli (Stimulus) influence individuals’ internal psychological states (Organism), which in turn trigger specific behavioral responses (Response). The S–O–R framework has been widely adopted in e-commerce and consumer behavior research. In this stream of literature, “stimuli” typically refer to external factors such as platform functionalities and product presentation cues; “organism” focuses on consumers’ psychological evaluations (e.g., perceived value and attitudinal responses); and “response” is commonly operationalized as purchase intention or actual purchase behavior.

As a novel consumption context characterized by virtual–real integration, AR-enabled e-commerce introduces distinctive technological features that serve as external stimuli fundamentally different from those in conventional online shopping environments. During AR-based shopping experiences, consumers’ perceived value and brand attitude emerge as critical psychological mechanisms linking these technological attributes to purchase intention. Compared with traditional e-commerce settings, the “stimulus” component in AR e-commerce is inherently immersive, interactive, and real-time in nature, which calls for a context-specific extension of the S–O–R framework. This constitutes the primary rationale for adopting the S–O–R paradigm as the core theoretical lens in the present study.

#### 3.1.2 Review of related studies and identification of research gaps.

**Technological attributes of AR e-commerce:** Existing research has examined certain technological characteristics of AR-enabled e-commerce, such as virtual try-on [14] and interactive product display [15], highlighting their positive effects on consumer experience. However, prior studies have largely investigated individual AR features in isolation. A systematic integration of four key AR e-commerce attributes—immersive virtual try-on, interactive product display, real-time fusion shopping, and personalized recommendation—remains limited. Moreover, the differentiated pathways through which these technological attributes affect consumer purchase intention have not been sufficiently clarified.

**Mediating roles of perceived value and brand attitude:** Perceived value has been consistently identified as a pivotal psychological driver of purchase intention in e-commerce contexts. AR technologies may enhance perceived value by increasing consumers’ sense of presence and improving the completeness and vividness of product information [16]. Brand attitude, in turn, can shape consumer decision-making by strengthening brand cognition, conveying brand meanings, and reinforcing brand-related evaluations [17]. Nevertheless, existing studies have not adequately elucidated how perceived value and brand attitude jointly mediate the relationship between AR technological attributes and purchase intention in AR-enabled shopping environments. In addition, the relative importance of these two mediators has rarely been empirically compared within a unified analytical framework.

**Application of the S–O–R framework in AR e-commerce:** Although some studies have attempted to apply the S–O–R framework to AR-related consumption scenarios, several limitations persist. First, AR e-commerce technological attributes have not been systematically theorized and operationalized as “stimulus” variables, resulting in insufficient theoretical alignment. Second, the transmission mechanism underlying the “stimulus–organism–response” process remains underexplored, and mediation effects are often not rigorously tested. Third, the empirical findings across studies are not fully consistent, which constrains the development of actionable and generalizable insights for practice.

In summary, prior research is characterized by limitations in theoretical adaptation, insufficient integration of multidimensional AR technological attributes, and inadequate examination of mediating mechanisms. To address these gaps, the present study develops and tests a multidimensional S–O–R model, aiming to provide a more systematic explanation of how AR e-commerce technological characteristics shape consumer purchase intention through key psychological pathways.

### 3.2 Research hypotheses

#### 3.2.1 AR e-commerce characteristics and perceived value.

Shopping experience, product information, personalized recommendations, and services are critical determinants of consumers’ perceived value. AR e-commerce allows consumers to interact with products, access detailed product information, and experience more immersive shopping. Personalized recommendations in AR e-commerce platforms cater to individual consumer needs.

According to Dacko (2017) [18], the effective application of AR technology enhances consumers’ sense of presence in the shopping process, improving their overall evaluation of products and services. Hilken et al. (2022) [19] found that virtual try-on experiences and interactive visual experiences in AR e-commerce improve the accuracy of product displays, enhancing consumers’ excitement and perceived value of AR platforms. Qin (2025) [20] examined the effects of AR experience on online purchase intention in terms of controllability, entertainment, responsiveness, and presentation quality.In retail, many AR e-commerce platforms provide personalized fashion experiences. For instance, IKEA allows users to virtually personalize interior design elements using AR, which enhances their perceived value and engagement in the shopping process [21].

Based on the above research, the following hypotheses are proposed:

H1: Immersive virtual try-on affects consumers’ perceived value.

H2: Interactive product presentations affects consumers’ perceived value.

H3: Real-time fusion shopping affects consumers’ perceived value.

H4: Personalized recommendations affects consumers’ perceived value.

#### 3.2.2 AR E-commerce characteristics and brand attitude.

AR technology enables consumers to engage in virtual try-on experiences, interactive presentations, and real-time fusion shopping, fostering direct interaction and connection with the brand. Scenario-based experiences on AR e-commerce platforms create consumer-brand connections by subtly conveying brand culture, building brand awareness, and enhancing brand image. These experiences can significantly influence consumers’ brand attitudes.

AR e-commerce platforms leverage AR technology to provide comprehensive product information, increasing brand awareness and improving brand attitudes [7]. Rauschnabel et al. (2019) [17] found that mobile AR apps can enhance brand attitudes by inspiring consumers, thereby strengthening the brand–consumer relationship. Lavoye et al. (2023) [22] found that virtual try-on services based on AR technology foster self-explorative engagement, which in turn enhances consumers’ brand recognition and positive brand-related outcomes. Smink et al. (2020) [23] demonstrated that augmented reality shopping environments enhance consumers’ perceived personalization, which in turn fosters more favorable brand and app evaluations compared to non-AR settings.

Based on these findings, the following hypotheses are proposed:

H5: Immersive virtual try-on affects consumers’ brand attitudes.

H6: Interactive product presentations affects consumers’ brand attitudes.

H7: Real-time fusion shopping affects consumers’ brand attitudes.

H8: Personalized recommendations affects consumers’ brand attitudes.

#### 3.2.3 Perceived value, brand attitude, and consumer purchase intention.

Perceived value serves as a key antecedent of consumer purchase intention, as it reflects consumers’ experiences and behaviors in specific contexts. Liu et al. (2021) [24] found that in social e-commerce environments, technological features indirectly influence consumers’ purchase intentions through a chain mediation of customer interaction and perceived value. Miao et al. (2022) [25] found that perceived value mediates the effect of e-customer satisfaction and e-trust on consumers’ repurchase intention in B2C e-commerce. Their findings suggest that when consumers feel satisfied and trust an e-commerce platform, they perceive more value, which in turn increases their likelihood of repurchasing. Similarly, Dong (2023) [26] demonstrated that brand attitude mediates the relationship between consumer identity and purchase intention, highlighting its crucial role in shaping consumers’ willingness to buy a brand. Guo and Zhang (2024) [27] conducted an empirical study examining how sensory, affective, cognitive, behavioral, and relational experiences in AR online shopping influence consumers’ purchase intentions, with perceived ease of use and perceived usefulness serving as mediating variables.

Based on the above discussion, the following research hypotheses are proposed:

H9: Perceived value affects purchase intention.

H10: Brand attitude affects purchase intention.

#### 3.2.4 The mediating role of perceived value and brand attitude.

Compared to traditional e-commerce, AR e-commerce enables consumers to intuitively understand a product’s appearance, size, texture, and other features through virtual try-on experiences. It also enhances interaction between consumers and products, providing deeper insights into product functionality and usage. Moreover, AR integrates virtual products into real-world environments, enabling consumers to observe real-time matching effects [28].

In terms of personalized consumption, AR technology enhances the shopping experience by offering customized product experiences and personalized recommendations through product interactions. The four characteristics of AR e-commerce act as external stimuli that influence purchase intentions via perceived value.

Based on the above discussion, the following research hypotheses are proposed:

H11: Perceived value mediates the relationship between immersive virtual try-on and consumer purchase intention.

H12: Perceived value mediates the relationship between interactive product presentation and consumer purchase intention.

H13: Perceived value mediates the relationship between real-time fusion shopping and consumer purchase intention.

H14: Perceived value mediates the relationship between personalized recommendations and consumer purchase intention.

AR technology enables e-commerce companies to enhance consumer-product interactions, fostering closer connections that shape brand image, improve brand attitude, and influence consumer behavior. AR online shopping features, such as virtual try-on experiences, product presentations, fusion shopping, and personalized recommendations, offer consumers greater product information value and an enhanced shopping experience. Higher perceived information value may strengthen consumers’ brand identity, thereby increasing purchase intention. A seamless interactive experience enhances brand favorability, further influencing consumers’ purchase decisions.

Based on the above discussion, the following research hypotheses are proposed:

H15: Brand attitude mediates the relationship between immersive virtual trials and consumer purchase intention.

H16: Brand attitude mediates the relationship between interactive product presentations and consumer purchase intention.

H17: Brand attitude mediates the relationship between real-time fusion shopping and consumer purchase intention.

H18: Brand attitude mediates the relationship between personalized recommendations and consumer purchase intention.

### 3.3 Model construction

Based on the research context and relevant research on AR e-commerce shopping, perceived value, brand attitude, and consumer purchase intention, this model integrates the S-O-R (Stimulus-Organism-Response) theory. It examines how consumers, after receiving external stimuli, form an overall evaluation of the product or service. Additionally, the emotional enjoyment or service experience provided by the product significantly influences perceived value.

**Fig 1:**
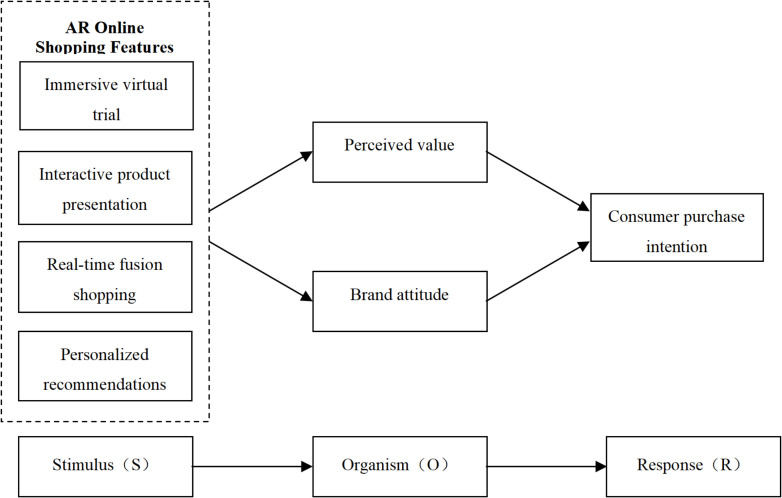
Theoretical model of factors influencing consumer purchase intention in AR e-commerce.

AR e-commerce shopping enhances consumer-brand interaction and connection, which significantly influences consumer attitudes. Consequently, perceived value and brand attitude are considered internal organism variables responding to external stimuli, while purchase intention is treated as the behavioral response variable. The theoretical model of factors influencing consumer purchase intention in AR e-commerce is illustrated in Fig 1.

### 3.4 Variable measurement

Based on a comprehensive review of relevant literature and in consideration of the research context and practical conditions, 24 observed variables were constructed to measure seven latent variables in the AR e-commerce consumer purchase intention model: Immersive Virtual Try-On, Interactive Product Display, Real-Time Fusion Shopping, Perceived Value, Brand Attitude, and Purchase Intention (see Table 2). A five-point Likert scale was employed to evaluate the measurement items, where scores of 1, 2, 3, 4, and 5 correspond to “strongly disagree,” “disagree,” “neutral,” “agree,” and “strongly agree,” respectively.

**Table 2:** Relevant variables and measurements.

Code	Latent Variable Name	|Observable Variable Code	Measurement Item	Source
IV	Immersive virtual trial	IV1	While using AR for shopping, I can fully focus on the virtual try-on experience.	[29] [30]
		IV2	The virtual try-on experience in AR shopping makes me feel enjoyable.	
		IV3	The virtual try-on experience in AR shopping engages me so deeply that I feel time passes very quickly.	
IP	Interactive product presentation	IP1	During AR shopping, I can freely rotate, zoom in, and zoom out the virtual products to examine their details, features, and functions.	[31] [32] [33]
		IP2	During AR shopping, I can freely move my body position and background to experience products from different angles and in various contexts.	
		IP3	During AR shopping, I have a certain degree of control over the virtual products presented by the AR mobile application/shopping website.	
RF	Real-time fusion shopping	RF1	During AR shopping, I can integrate virtual products into real environments in real time, achieving a fusion of the virtual and the real.	This study
		RF2	During AR shopping, I can observe product appearance and matching effects in real time, and assess how well the product fits with the physical environment.	
		RF3	The AR e-commerce shopping website has sufficient processing capacity and response speed to provide me with real-time product experiences.	
VP	Personalized recommendations	VP1	The AR e-commerce shopping website provides products and services that meet my specific needs.	[34]
		VP2	The AR e-commerce shopping website offers personalized product recommendations based on my preferences and past purchase history.	
		VP3	The AR e-commerce shopping website pays close attention to customer needs.	
PV	Perceived value	PV1	The products and services provided by the AR e-commerce shopping website make me feel that they are worth the value.	[35]
		PV2	The AR e-commerce shopping website helps me quickly obtain more product information within a short time, thereby improving my shopping efficiency.	
		PV3	The AR e-commerce shopping website is novel and brings me a sense of freshness, which attracts me to shop.	
		PV4	During AR shopping, I feel relaxed and happy, which enhances my overall shopping experience.	
BA	Brand attitude	BA1	I have a very favorable impression of this brand.	[36]
		BA2	I believe the products and services of this brand are more satisfactory.	
		BA3	Compared with other similar brands, I am more interested in this brand.	
		BA4	Compared with other similar brands, I am more likely to choose this brand.	
PI	Purchasing intention	PI1	When I have a need, I am willing to purchase products on the AR e-commerce shopping website.	[37]
		PI2	The features of the AR e-commerce shopping website are valuable references for my purchase decisions.	
		PI3	I am willing to recommend the AR e-commerce shopping website to people around me.	
		PI4	In the future, I am more willing to purchase the products I like through the AR e-commerce shopping website.	

## 4. Empirical analysis of factors influencing consumer purchase intention in AR e-commerce

### 4.1 Descriptive statistics of the sample

The survey targeted users who had experience with or were familiar with AR e-commerce shopping. Before data collection, a questionnaire link was generated using the Wenjuanxing platform. To ensure that respondents completed the survey conscientiously, screening items with predetermined correct options were included. In addition, restrictions were applied to allow only one submission per IP address, thereby ensuring the quality of the collected data. Before completing the questionnaire, all participants read and signed an electronic informed consent form. Participants were fully informed of the research purpose, data usage, and anonymity principles, and participation in the survey was voluntary. The present study strictly adhered to academic ethical standards. Before completing the questionnaire, all participants read and signed an electronic informed consent form and were fully informed of the research purpose, data usage, and anonymity principles before voluntarily participating in the survey.

A total of 527 questionnaires were collected through multiple channels, including WeChat, QQ, and the sample service function provided by Wenjuanxing. After excluding four categories of invalid questionnaires—those from respondents without AR shopping experience or familiarity, those completed in less than one minute, those with identical answers across all items, and those with incorrect responses to the screening items—482 valid questionnaires were retained. The effective response rate was 91.46%. The basic characteristics of the sample are presented in Table 3.

**Table 3:** Basic characteristics of the sample (N = 482).

Variable	Option	Number of People	Proportion (%)	Variable	Option	Number of People	Proportion (%)
Gender	Male	194	40.2	Do you know about AR online shopping?	Know and have used	444	92.1
	Female	288	59.8		Know but have never used	38	7.9
Age	Under 18	6	1.2	Which AR mobile apps/websites have you used? (Multiple selections)	Taobao	241	23.3%
	18–30	241	50.0		JD.com	318	30.7%
	31–50	192	39.8		Dewu	301	29.1%
	Over 50	43	8.9		IKEA	62	6.0%
Education Level	High school or below	69	14.3		Amazon	91	8.8%
	Associate degree	164	34.0		Other	23	2.2%
	Bachelor’s degree	176	36.5	What types of products do you typically buy through AR apps/websites? (Multiple selections)	Furniture	62	7.2%
	Master’s degree or above	73	15.1		Shoes	329	37.9%
Monthly Income	Below 3,000 yuan	58	12.0		Cosmetics	288	33.2%
	3,000–6,000 yuan	144	29.9		Appliances	69	8.0%
	6001 ~ 9000yuan	166	34.4		Watches	62	7.2%
	9001 ~ 12000yuan	51	10.6		Other	57	6.4%
	12,001–20,000 yuan	40	8.3				
	Above 20,000 yuan	23	4.8				

### 4.2 Reliability and validity analysis

Reliability refers to the assessment of a questionnaire’s consistency, reflecting the degree to which repeated measurements of the same object with the same method yield consistent results. In this study, Cronbach’s α coefficient was employed to assess reliability, with higher values indicating greater reliability. SPSS 26.0 was used to analyze both the overall reliability of the questionnaire and the reliability of each variable.

The questionnaire consisted of 24 items, and the overall Cronbach’s α coefficient was 0.946, indicating strong reliability of the scale. For the seven variables—Immersive Virtual Try-On, Interactive Product Display, Real-Time Fusion Shopping, Personalization, Perceived Value, Brand Attitude, and Consumer Purchase Intention—the Cronbach’s α coefficients ranged from 0.856 to 0.915, all exceeding the 0.8 threshold. These results demonstrate that the reliability of each variable meets the required standard (see Table 4).

**Table 4:** Reliability values of each variable in the questionnaire.

Variable Name	Number of Items	Cronbach’s α Coefficient
Immersive virtual trial	3	0.877
Interactive product presentation	3	0.856
Real-time fusion shopping	3	0.858
Personalized recommendations	3	0.875
Perceived value	4	0.914
Brand attitude	4	0.908
Purchasing intention	4	0.915

Convergent validity refers to the degree of correlation among measurement items within the same factor. It is assessed using standardized factor loadings (Std. Estimate), composite reliability (CR), and average variance extracted (AVE). As shown in Table 5, the standardized factor loadings of the measurement items for Immersive Virtual Try-On, Interactive Product Display, Real-Time Fusion Shopping, Personalization, Perceived Value, Brand Attitude, and Consumer Purchase Intention were all greater than 0.7, indicating that the items adequately represent their respective latent variables. The AVE values of all latent variables exceeded 0.6, and the CR values were greater than 0.8. Therefore, the questionnaire demonstrates satisfactory convergent validity and is suitable for subsequent analysis.

**Table 5:** Convergent validity test.

Variable	Item	Standardized Factor Loadings	CR	AVE
Immersive virtual trial	IV1	0.826	0.879	0.708
	IV2	0.787		
	IV3	0.906		
Interactive product presentation	IP1	0.828	0.857	0.667
	IP2	0.773		
	IP3	0.848		
Real-time fusion shopping	RF1	0.838	0.859	0.670
	RF2	0.792		
	RF3	0.824		
Personalized recommendations	VP1	0.856	0.876	0.701
	VP2	0.797		
	VP3	0.866		
Perceived value	PV1	0.811	0.913	0.725
	PV2	0.869		
	PV3	0.831		
	PV4	0.893		
Brand attitude	BA1	0.792	0.908	0.711
	BA2	0.860		
	BA3	0.828		
	BA4	0.890		
Purchasing intention	PI1	0.809	0.914	0.728
	PI2	0.886		
	PI3	0.849		
	PI4	0.866		

Discriminant validity refers to the degree of distinction among measurement items of different factors. In this study, it was assessed by comparing the square root of the average variance extracted (AVE) with the correlation coefficients among the variables. As shown in Table 6, the square roots of the AVE values on the diagonal were all greater than the correlation coefficients between the variables, indicating that the questionnaire possesses good discriminant validity.

**Table 6:** Discriminant validity test.

	PI	BA	PV	VP	RF	IP	IV
PI	0.853						
BA	0.629	0.843					
PV	0.590	0.588	0.851				
VP	0.538	0.596	0.548	0.837			
RF	0.581	0.591	0.592	0.525	0.819		
IP	0.473	0.534	0.535	0.470	0.558	0.817	
IV	0.527	0.504	0.514	0.509	0.481	0.502	0.841

### 4.3 Correlation analysis

Correlation analysis examines the relationships and degrees of association between variables. This study employs the Pearson method to assess correlations between variables. Correlations were observed between the four characteristics of AR e-commerce and perceived value, brand attitude, and consumer purchase intention, with all correlation coefficients below 0.75. These results indicate that multicollinearity is not present (see Table 7).

**Table 7:** Correlation analysis.

	IV	IP	RF	VP	PV	BA	PI
IV	1						
IP	.551**	1					
RF	.500**	.543**	1				
VP	.530**	.475**	.554**	1			
PV	.444**	.516**	.543**	.501**	1		
BA	.505**	.476**	.543**	.560**	.529**	1	
PI	.520**	.456**	.493**	.526**	.538**	.574**	1

Note: **Significantly correlated at the 0.01 level (two-tailed).

### 4.4 Structural equation model analysis

**Fig 2:**
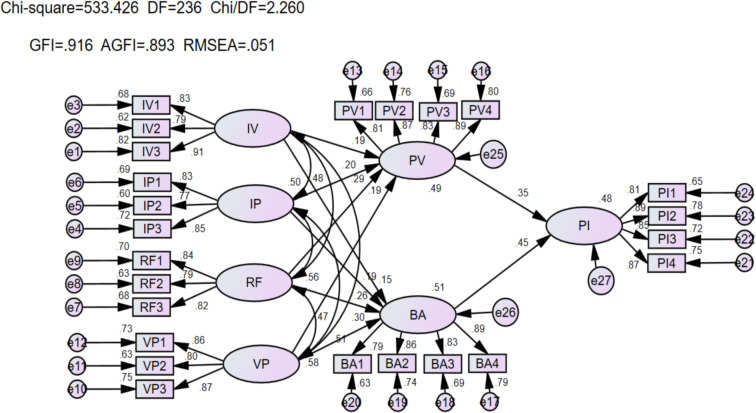
Standardized results of the structural equation model analysis.

AMOS 26.0 was used to conduct structural equation modeling with four exogenous latent variables—Immersive Virtual Try-On (IV), Interactive Product Display (IP), Real-Time Fusion Shopping (RF), and Personalization (VP)—and three endogenous latent variables—Perceived Value (PV), Brand Attitude (BA), and Consumer Purchase Intention (PI). The results are presented in Fig 2.

The overall fit indices obtained from AMOS 26.0 analysis are presented in Table 8. The CMIN/DF value of 2.260 satisfies the requirement of being less than 3. The GFI, NFI, CFI, and IFI indices all exceed the threshold of 0.9, while the RMSEA value of 0.051 is near the ideal 0.05. All fit indices fall within acceptable ranges, indicating a good model fit.

**Table 8:** Overall fit indices of the structural equation model.

Indicator	CMIN/DF	RMSEA	GFI	NFI	CFI	IFI
Acceptable Standard	≤5	≤0.08	≥0.9	≥0.9	≥0.9	≥0.9
Ideal Standard	≤3	≤0.05	The closer to 1, the better	The closer to 1, the better	The closer to 1, the better	The closer to 1, the better
Actual Fit Value	2.260	0.051	0.916	0.938	0.964	0.964
Meets Requirement or Not	Yes	Acceptable	Yes	Yes	Yes	Yes

The significance of the standardized path coefficients is assessed using the C.R. value and the P-value. A C.R. value greater than 1.96 and a P-value less than 0.05 indicate a significant path effect between variables, supporting the corresponding hypothesis. Based on the C.R. values and P-values, all standardized path coefficients were statistically significant. The standardized path coefficients of the structural equation model and the hypothesis tests are presented in Table 9.

**Table 9:** Standardized path coefficients and test results of the model.

Hypothesis Number	Path	Standardized Path Coefficient	C.R.	P-value	Test Result
H1	Immersive Virtual Trial → Perceived Value	0.187	3.684	***	Confirmed
H2	Interactive product presentation → Perceived Value	0.196	3.621	***	Confirmed
H3	Real-time fusion shopping → Perceived Value	0.290	4.933	***	Confirmed
H4	Immersive Virtual Trial → Brand Attitude	0.149	2.991	0.003	Confirmed
H5	Interactive product presentation → Brand Attitude	0.186	3.523	***	Confirmed
H6	Real-time fusion shopping → Brand Attitude	0.257	4.500	***	Confirmed
H7	Personalized recommendations → Brand Attitude	0.295	5.482	***	Confirmed
H8	Personalized recommendations → Perceived Value	0.193	3.554	***	Confirmed
H9	Perceived Value → Consumer Purchase Intention	0.347	7.342	***	Confirmed
H10	Brand Attitude → Consumer Purchase Intention	0.449	9.437	***	Confirmed

Note: *** indicates P < 0.001.

Empirical results indicate that the immersive virtual try-on feature of AR e-commerce has a significant positive effect on consumers’ perceived value (β = 0.187, p < 0.001) and brand attitude (β = 0.149, p < 0.01). Thus, H1 and H4 are supported, suggesting that immersive virtual try-on experiences positively influence perceived value and brand attitude. The interactive product display feature of AR e-commerce has a significant positive effect on perceived value (β = 0.196, p < 0.001) and brand attitude (β = 0.186, p < 0.001). Accordingly, H2 and H5 are supported, indicating that effective interactive product displays enhance both perceived value and brand attitude.

The real-time fusion shopping feature of AR e-commerce has a significant positive effect on perceived value (β = 0.290, p < 0.001) and brand attitude (β = 0.257, p < 0.001). Thus, H3 and H6 are supported, showing that real-time visualization experiences positively shape consumers’ perceived value and brand attitude. Furthermore, the personalization feature of AR e-commerce demonstrates a significant positive effect on perceived value (β = 0.193, p < 0.001) and brand attitude (β = 0.295, p < 0.001). Hence, H7 and H8 are supported, indicating that personalized recommendations enhance perceived value and brand attitude.

Finally, consumers’ perceived value (β = 0.347, p < 0.001) and brand attitude (β = 0.449, p < 0.001) exert significant positive effects on purchase intention. Therefore, H9 and H10 are supported, confirming that perceived value and brand attitude play critical roles in promoting consumers’ purchase intentions.

### 4.5 Mediation effect test

If a direct effect exists between the independent and dependent variables, it is further necessary to examine whether the independent variable significantly influences the mediator and whether the mediator significantly affects the dependent variable. If both paths are significant, the significance of the indirect regression coefficient is tested. A significant coefficient indicates that the mediator plays a partial mediating effect, whereas a non-significant coefficient suggests a full mediating effect.

To investigate the relationships among immersive virtual try-on, interactive product display, real-time fusion shopping, personalization, and consumers’ purchase intention in AR online shopping, this study applied the Bootstrap method to test the mediating roles of perceived value and brand attitude. The PROCESS macro (Hayes, 2013) was employed with 5,000 bootstrap resamples. At the 95% confidence level, if the confidence interval does not include zero, the mediating effect is considered significant.

(1) Mediating Effect of Perceived Value

The test of perceived value as a mediator between immersive virtual try-on and purchase intention produced a 95% confidence interval of [0.067, 0.197], which did not include zero, confirming a significant mediating effect (effect size = 0.127). After controlling for perceived value, the direct effect of immersive virtual try-on on purchase intention remained significant, indicating that perceived value plays a partial mediating role in this relationship. Thus, H11 is supported.

Applying the same procedure, perceived value was also found to partially mediate the relationships between interactive product display and purchase intention (95% CI [0.087, 0.233], effect size = 0.159), real-time fusion shopping and purchase intention (95% CI [0.085, 0.237], effect size = 0.156), and personalization and purchase intention (95% CI [0.074, 0.211], effect size = 0.137). Accordingly, H12, H13, and H14 are supported.

(2) Mediating Effect of Brand Attitude

Using the same method, brand attitude was tested as a mediator between each independent variable and purchase intention. The results indicate that brand attitude partially mediates the associations between immersive virtual try-on (95% CI [0.102, 0.251], effect size = 0.169), interactive product display (95% CI [0.125, 0.284], effect size = 0.194), real-time fusion shopping (95% CI [0.127, 0.283], effect size = 0.200), and personalization (95% CI [0.110, 0.266], effect size = 0.182) and purchase intention. These findings confirm H15, H16, H17, and H18. Detailed results are reported in Table 10.

**Table 10:** Mediation effect test results.

Hypothesis Number	Mediation Path	Indirect Effect Value	95% Confidence Interval		Main Effect After Controlling for Mediator	Path Result
			Lower Limit	Upper Limit		
H11	Immersive Virtual Trial → Perceived Value → Consumer Purchase Intention	0.127	0.067	0.197	Significant	Partial Mediation
H12	Interactive product presentation → Perceived Value → Consumer Purchase Intention	0.159	0.087	0.233	Significant	Partial Mediation
H13	Real-time fusion shopping→ Perceived Value → Consumer Purchase Intention	0.156	0.085	0.237	Significant	Partial Mediation
H14	Personalized recommendations→Perceived Value → Consumer Purchase Intention	0.137	0.074	0.211	Significant	Partial Mediation
H15	Immersive Virtual Trial→ Brand Attitude → Consumer Purchase Intention	0.169	0.102	0.251	Significant	Partial Mediation
H16	Interactive product presentation→ Brand Attitude → Consumer Purchase Intention	0.194	0.125	0.284	Significant	Partial Mediation
H17	Real-time fusion shopping→ Brand Attitude → Consumer Purchase Intention	0.200	0.127	0.283	Significant	Partial Mediation
H18	Personalized recommendations→Brand Attitude → Consumer Purchase Intention	0.182	0.110	0.266	Significant	Partial Mediation

The overall hypothesis testing results for both direct effects and mediation effects are shown in Table 11.

**Table 11:** Hypothesis testing results.

Hypothesis Number	Hypothesis Path Description	Test Result
H1	Immersive virtual trial has a positive impact on consumer perceived value.	Confirmed
H2	Interactive product presentation has a positive impact on consumer perceived value.	Confirmed
H3	Real-time fusion shopping has a positive impact on consumer perceived value.	Confirmed
H4	Personalized recommendations have a positive impact on consumer perceived value.	Confirmed
H5	Immersive virtual trial has a positive impact on consumer brand attitude.	Confirmed
H6	Interactive product presentation has a positive impact on consumer brand attitude.	Confirmed
H7	Real-time fusion shopping has a positive impact on consumer brand attitude.	Confirmed
H8	Personalized recommendations have a positive impact on consumer brand attitude.	Confirmed
H9	Perceived value has a positive impact on consumer purchase intention.	Confirmed
H10	Brand attitude has a positive impact on consumer purchase intention.	Confirmed
H11	Consumer perceived value mediates the relationship between immersive virtual trial and purchase intention.	Partial Mediation
H12	Consumer perceived value mediates the relationship between Interactive product presentation and purchase intention.	Partial Mediation
H13	Consumer perceived value mediates the relationship between Real-time fusion shopping and purchase intention.	Partial Mediation
H14	Consumer perceived value mediates the relationship between Personalized recommendations and purchase intention.	Partial Mediation
H15	Consumer brand attitude mediates the relationship between immersive virtual trial and purchase intention.	Partial Mediation
H16	Consumer brand attitude mediates the relationship between interactive product presentation and purchase intention.	Partial Mediation
H17	Consumer brand attitude mediates the relationship between Real-time fusion shopping and purchase intention.	Partial Mediation
H18	Consumer brand attitude mediates the relationship between Personalized recommendations and purchase intention.	Partial Mediation

## 5. Influencing factors and mechanisms

### 5.1 Influencing factors

(1) Effects of AR E-Commerce Features on Perceived Value and Brand Attitude

Compared with offline shopping, traditional online shopping offers limited cues for evaluating whether products meet consumers’ needs. Reliance on images, short videos, or live streams often leads to discrepancies between expectations and actual product experiences, thereby diminishing satisfaction. In contrast, AR-enabled shopping provides immersive virtual try-on and product trial experiences, allowing consumers to directly visualize how products look and perform in real-life contexts.. These immersive features substantially increase perceived value and foster more favorable brand attitudes.

Interactive product display is another critical mechanism. Unlike static two-dimensional presentations, AR’s advanced rendering and visualization capabilities create realistic 3D product models that vividly present appearance, texture, and internal structure. Consumers can freely rotate, zoom in on, and manipulate these models to examine details from multiple perspectives. This enhanced interactivity deepens product understanding, improves decision-making confidence, and strengthens consumers’ perceived connection with the product, thereby enhancing both perceived value and brand attitudes.

Furthermore, AR empowers real-time fusion shopping, enabling consumers to place virtual products such as furniture, appliances, or decorative items into their physical environments. This integration allows them to evaluate compatibility in terms of size, style, and color in real time, significantly enhancing experiential value. Personalized AR functions—such as customizing product colors, materials, or configurations—further enrich the experience. Tailored recommendations and product-matching services aligned with consumer preferences increase satisfaction and loyalty, thereby reinforcing positive brand attitudes [29,31].

(2) Mediating Roles of Perceived Value and Brand Attitude in Purchase Intention

In AR e-commerce, perceived value and brand attitude serve as key mediating mechanisms linking AR features to purchase intention. When consumers perceive high value—through enriched product information, immersive experiences, personalization, and reliable service—their expectations are more fully met, which strengthens willingness to purchase [38].

Brand attitude also functions as a pivotal antecedent of purchase intention. Stronger recognition, trust, and emotional attachment toward a brand translate into greater willingness to engage in repeated transactions. When AR-enabled shopping is perceived as engaging, informative, and satisfying, consumers’ perceived value and brand attitudes are elevated. These enhanced psychological states, in turn, mediate the relationship between AR features and purchase intention, confirming that both constructs are critical drivers of consumer decision-making [39].

(3) Direct Effects of AR Features on Purchase Intention

Beyond mediating pathways, AR e-commerce features directly influence consumer purchase intentions. As online shoppers increasingly prioritize experiential quality, AR creates a new “human–product–environment” interaction scenario that delivers richer product information and offline-like experiences. The more immersive the shopping experience, the stronger consumers’ visual and tactile engagement, which stimulates purchase interest and motivates buying behavior.

Greater interactivity fosters a stronger sense of involvement and recognition, reinforcing purchase intentions. Real-time fusion shopping provides vivid, context-specific product visualizations that increase the accuracy of product information, thereby enhancing trust, emotional reliance, and brand loyalty. Similarly, personalized recommendations that align with consumer preferences intensify brand affinity and strengthen purchase intentions. Collectively, these mechanisms confirm AR’s role as a transformative driver of consumer behavior in digital commerce [12].

### 5.2 Mechanism of action

(1) The Impact of AR E-Commerce Features on Perceived Value and Brand Attitude

Compared to offline shopping, traditional online shopping do not allow consumers to fully assess whether a product meets their needs. Consumers often rely on images, short videos, or live streams to infer a product’s functionality. However, discrepancies between expectations and reality upon receiving the product can negatively impact the shopping experience [31].

In contrast, AR e-commerce provides virtual try-on and product trial experiences, allowing consumers to experience how the product looks and feels. This enhances perceived value and fosters a more favorable brand attitude [40].

Interactive product displays significantly influence consumers’ perceived value and brand attitude. Unlike traditional 2D images, AR technology offers advanced visualization capabilities. High-level rendering functions produce realistic visual effects, allowing 3D models to vividly showcase a product’s appearance, texture, and internal structure [7]. Consumers can freely rotate, zoom in, and zoom out on virtual products, examining details and features from every angle. This interactive process enhances consumer understanding of product characteristics and benefits, boosting confidence in purchase decisions.

The interactive product display feature of AR online shopping overcomes the limitations of traditional methods, fostering a dynamic connection between consumers and products. This enhances the overall shopping experience and perceived engagement.

AR technology integrates online and offline environments, creating a seamless shopping experience. It generates 3D effects for items like furniture, appliances, or decorative artworks, allowing consumers to visualize how the product would look in use on their screens. This capability accurately reproduces product dimensions and proportions, enabling consumers to freely place items in their homes and observe their compatibility with real-world environments. This interaction significantly boosts perceived value throughout the shopping experience.

Integrating online and offline services also strengthens brand recognition and affinity, leading to a more positive brand attitude. AR e-commerce enables highly personalized experiences, allowing consumers to design layouts, match products, adjust colors, and modify materials or shapes to meet their preferences [38]. Personalized recommendations, product pairings, and usage recommendations tailored to consumer needs enhance satisfaction and enrich the shopping experience. These factors foster greater brand loyalty and attachment, contributing to a more favorable attitude toward the brand.

(2) The Role of Perceived Value and Brand Attitude in Consumer Purchase Intentions

In AR e-commerce shopping, when consumers perceive high value in a product or service, their needs and expectations are better satisfied, thereby strengthening their purchase intentions. Perceived value includes key factors such as shopping experience, product information, personalization, and service quality. The application of AR technology in online shopping enhances the shopping experience and increases consumers’ perceived value of products and services. When consumers exhibit strong recognition and affinity for a brand, they are more likely to trust and prefer that brand, further boosting their purchase intentions [41].

Perceived value and brand attitude serve as mediators between AR online shopping features and consumer purchase intentions. Consumers are more likely to recognize and accept a brand’s products and services when they find them interesting, satisfying, engaging, and informative, or when the provided information is perceived as accurate, reliable, and useful [29]. This recognition and acceptance, reflected in perceived value and brand attitude, ultimately influence their purchase intentions.

(3) The Impact of AR E-Commerce Features on Consumer Purchase Intentions

Consumers are increasingly focused on the experiential aspects of online shopping and seek comprehensive, detailed product information when selecting products. AR technology in online shopping creates a new “human-product” interaction scenario. It allows consumers to experience a near-offline shopping environment and access detailed product information. The more immersive the shopping experience, the more it enhances consumers’ visual and tactile perceptions, sparking their interest and motivating them to make a purchase [2].

Greater product interactivity increases consumers’ involvement and perceived value during the online shopping process, leading to stronger purchase intentions. The real-time fusion shopping feature presents products in real-world settings with vivid visualizations. High-quality visualizations provide accurate product information, boosting consumer trust in the brand. This trust fosters emotional attachment and brand loyalty, ultimately enhancing purchase intentions.

Additionally, when a brand or retailer better meets consumers’ needs and preferences, the perceived quality of personalized service improves. This improvement leads to higher brand affinity and stronger purchase intentions [7].

### 5.3 Countermeasures and recommendations

(1) Highlighting Key Performance Factors of AR E-Commerce

Although AR e-commerce is gaining visibility and acceptance, particularly among younger consumers, the rapid iteration of digital technologies shortens the lifecycle of applications and leaves consumers less time to adapt. To maintain competitiveness, businesses must keep pace with technological development, enhance consumer satisfaction, and mitigate uncertainties associated with technological change. The core appeal of AR shopping lies in its ability to provide experiences unavailable in traditional formats. Merchants should therefore prioritize optimizing performance factors that directly shape consumer perceptions, such as the completeness and accuracy of product information, as well as the usability and smoothness of AR systems. By improving these dimensions, firms can reinforce consumers’ perceived value and brand attitude—both of which are decisive drivers of purchase intention.

In immersive virtual try-on, firms should invest in advanced modeling and rendering technologies to ensure precise alignment between virtual products and consumers’ physical features, delivering a more realistic and immersive try-on experience. For interactive product displays, merchants should improve texture and material rendering to enhance detail and realism, while ensuring information accuracy and comprehensiveness. Interface design should also emphasize usability and convenience, accommodating consumer preferences and behaviors. Real-time feedback loops should be established to continuously refine user interaction and optimize satisfaction [21].

In real-time fusion shopping, greater investment in image recognition and tracking algorithms is necessary to enhance accuracy and responsiveness under complex conditions such as low light. Improved integration between virtual products and real environments can generate more authentic visualizations, strengthening consumers’ perceived value. Achieving precise alignment of “scenario–experience–consumption” fosters a closed-loop transaction system, enhancing satisfaction and building stronger brand goodwill.

(2) Strengthening Content Quality in AR E-Commerce

Despite the value of technological innovation, product quality and utility remain the primary determinants of consumer trust. AR e-commerce firms must therefore not only enrich consumer experiences and provide detailed product information but also ensure that product and service quality consistently meets expectations. A positive impression of product quality and service reliability enhances both consumer satisfaction and platform credibility.

Equally important are timely and responsive pre-sales, in-sales, and after-sales services. Businesses should address consumer inquiries promptly and provide professional responses to negative reviews. Proactive reputation management not only demonstrates accountability to individual customers but also signals sincerity and reliability to broader audiences, thereby reinforcing trust and loyalty.

(3) Enhancing User Sharing and Community Mechanisms

Although AR shopping primarily involves individual experiences such as virtual try-on and fusion shopping, social influence plays a critical role in adoption and diffusion. Firms should promote social synergy by integrating AR shopping platforms with social media channels (e.g., Xiaohongshu, TikTok/Douyin), encouraging users to share experiences and amplifying the reach and influence of AR shopping.

Intelligent algorithms can identify influential consumers or key opinion leaders (KOLs) to leverage network effects. Reward-based mechanisms may incentivize referrals and sharing behaviors, strengthening user acquisition through peer influence. Beyond individual sharing, firms should establish brand-centered virtual communities to foster interaction, emotional connection, and knowledge exchange among consumers [42]. Organizing exclusive brand activities within these communities enhances participation and nurtures belonging, which strengthens brand loyalty and long-term engagement.

(4) Upgrading Privacy Protection in AR E-Commerce

AR e-commerce requires extensive consumer data, raising privacy and security concerns that may undermine trust. For example, facial data collected during virtual makeup trials could cause serious consumer distress if misused. The massive, heterogeneous, and easily accessible nature of digital data creates vulnerabilities, while regulatory frameworks and anonymization practices remain insufficient.

To address these challenges, AR e-commerce firms must adopt multi-dimensional strategies to strengthen privacy governance. This includes robust data protection systems, stricter monitoring against leakage and misuse, and safeguards ensuring lawful data use. By guaranteeing security and reliability, firms can provide personalized services without compromising user privacy [15]. Such measures alleviate consumer concerns, increase trust, and enhance willingness to engage in AR-enabled shopping.

## 6. Conclusions and implications

### 6.1 Conclusions

Based on 482 valid questionnaire responses, this study develops and tests an AR-enabled e-commerce purchase intention model grounded in the stimulus–organism–response (S–O–R) framework. The findings yield three key conclusions. First, the four technological attributes of AR e-commerce—immersive virtual try-on, interactive product display, real-time fusion shopping, and personalized recommendation—exert significant and positive effects on both perceived value and brand attitude. Second, perceived value and brand attitude play partial mediating roles in the relationships between AR technological attributes and consumers’ purchase intention, indicating that technological features shape purchase intention partly through consumers’ psychological evaluations. Third, the effect of real-time fusion shopping on perceived value is the strongest among the four AR attributes, whereas personalized recommendation demonstrates the most salient impact on brand attitude. Moreover, brand attitude exhibits a stronger direct influence on purchase intention than perceived value, highlighting the pivotal role of attitudinal responses in AR-based purchase decision-making.

### 6.2 Theoretical and practical implications

#### 6.2.1 Theoretical implications.

This study offers several theoretical implications. First, it extends the applicability of the S–O–R framework to virtual–real integrated consumption settings, thereby contributing to a more context-sensitive understanding of consumer behavior in the digital economy. Second, by clarifying the psychological pathways through which AR technological attributes translate into purchase intention, this research enriches the technology marketing literature and provides a more mechanism-based explanation of consumer responses to AR-enabled shopping experiences. Third, the multidimensional operationalization of AR e-commerce attributes offers a standardized set of “stimulus” dimensions for future empirical research, facilitating more systematic and comparable investigations into AR-based consumption scenarios.

#### 6.2.2 Practical implications.

The findings also provide actionable implications for practitioners.

Implications for e-commerce platforms. Platforms should prioritize technological investment to enhance the contextual adaptability of real-time fusion shopping, particularly by improving the accuracy of image recognition and tracking systems to ensure a seamless alignment between virtual products and real-world environments. In addition, platforms are encouraged to refine personalized recommendation algorithms by leveraging consumers’ browsing patterns, purchase histories, and preference signals to improve recommendation relevance and effectiveness. Finally, user interface design should be optimized to simplify AR interaction processes and reduce consumers’ perceived effort and technical barriers, thereby improving the overall usability of AR shopping functions [16].

Implications for brands. Brands should focus on strengthening immersive virtual try-on and interactive product display functionalities by adopting high-fidelity 3D modeling and visualization techniques that improve the consistency between virtual experiences and offline product realities. Furthermore, AR-based experiences can be strategically used to convey brand values and cultural meanings, enabling consumers to develop stronger brand identification through personalized engagement. Brands may also establish an integrated service mechanism linking AR experience and after-sales support, allowing timely responses to consumer concerns during the AR experience and ultimately reinforcing brand reputation.

Implications for the industry. At the industry level, stakeholders should consider establishing technical standards and privacy protection guidelines for AR e-commerce, clarifying the boundaries of data collection, storage, and usage to safeguard consumer privacy. Moreover, integrating AR-enabled shopping with social media platforms can encourage consumers to share AR experiences, thereby amplifying social diffusion effects and enhancing the market influence of AR e-commerce. Finally, cross-industry collaborations should be promoted to expand AR applications into additional product categories (e.g., fresh food and automobiles), which would broaden the boundaries and scalability of AR-enabled commerce.

### 6.3 Theoretical contributions and limitations

#### 6.3.1 Theoretical contributions.

The present study contributes to the literature in three primary ways. First, it systematically conceptualizes and operationalizes four core technological attributes of AR-enabled e-commerce—immersive virtual try-on, interactive product display, real-time fusion shopping, and personalized recommendation—as “stimulus” dimensions within the S–O–R framework. By doing so, it addresses the theoretical adaptation gap of the S–O–R paradigm in virtual–real integrated consumption contexts and expands its boundary conditions. Second, the present study proposes an integrated analytical framework linking technological attributes, psychological perceptions, and purchase intention, and empirically validates the dual mediating roles of perceived value and brand attitude. This contributes to a more refined understanding of the “technology–scenario–behavior” transmission mechanism in AR e-commerce and offers a theoretically grounded perspective for future research on digital consumption contexts. Third, by comparing the differentiated effects of multiple AR technological attributes, the study identifies the particularly influential roles of real-time fusion shopping and personalized recommendation, thereby addressing the limited evidence in prior studies regarding the synergistic and heterogeneous impacts of multidimensional AR features.

#### 6.3.2 Limitations and future research directions.

Despite its contributions, the present study has several limitations. First, the sample was collected primarily through online channels, which may introduce sampling bias in terms of demographic distribution (e.g., age structure) and consumption patterns. Future research may combine online and offline data collection approaches to enhance sample representativeness and external validity. Second, the present study does not incorporate potential moderating factors related to consumer heterogeneity, such as technology readiness, risk preference, or privacy concerns. Future studies are encouraged to include moderating variables to further enrich the explanatory power and boundary conditions of the proposed model.

## Supporting information

S1 Data Data. (XLSX)
